# Hierarchical Composite Self‐Sorted Supramolecular Gel Noodles

**DOI:** 10.1002/adma.202211277

**Published:** 2023-03-15

**Authors:** Libby J. Marshall, Matthew Wallace, Najet Mahmoudi, Giuseppe Ciccone, Claire Wilson, Massimo Vassalli, Dave J. Adams

**Affiliations:** ^1^ School of Chemistry University of Glasgow Glasgow G12 8QQ UK; ^2^ School of Pharmacy University of East Anglia Norwich Research Park Norwich NR4 7TJ UK; ^3^ ISIS Neutron and Muon Source Rutherford Appleton Laboratory Didcot OX11 0QX UK; ^4^ Centre for the Cellular Microenvironment Advanced Research Centre University of Glasgow Glasgow G11 6EW UK

**Keywords:** hydrogels, multicomponent systems, self‐sorting, supramolecular structures

## Abstract

Multicomponent supramolecular systems can be used to achieve different properties and new behaviors compared to their corresponding single component systems. Here, a two‐component system is used, showing that a non‐gelling component modifies the assembly of the gelling component, allowing access to co‐assembled structures that cannot be formed from the gelling component alone. The systems are characterized across multiple length scales, from the molecular level by NMR and CD spectroscopy to the microstructure level by SANS and finally to the material level using nanoindentation and rheology. By exploiting the enhanced mechanical properties achieved through addition of the second component, multicomponent noodles are formed with superior mechanical properties to those formed by the single‐component system. Furthermore, the non‐gelling component can be triggered to crystallize within the multicomponent noodles, allowing the preparation of new types of hierarchical composite noodles.

## Graphical abstract

Multicomponent assembly of 2NapFF with the structurally similar functionalized dipeptide 2NapLG causes changes to the formed first‐order supramolecular structures. These changes to assembly allow access to new properties and behaviors. This multicomponent system can be used to form mechanically robust supramolecular noodles. Through a change in external conditions (a reduction in pH), crystals can be formed within these noodles.

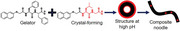

## 1 Introduction

**Figure 1:**
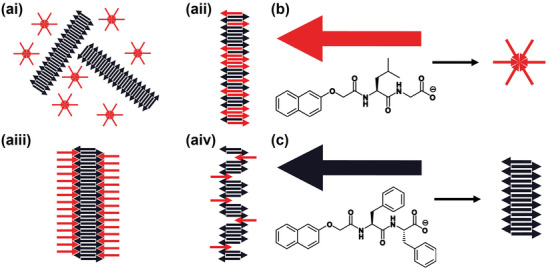
a) Schematic representations of: i) self‐sorting, ii) co‐operative co‐assembly iii) orthogonal co‐assembly, and iv) disruptive co‐assembly in a two‐component system. b,c) Chemical structures of the two components studied here: b) 2NapLG (shown schematically as a red arrow) which self‐assembles to form non‐persistent micelles at high pH and c) 2NapFF (shown schematically as a black arrow) which self‐assembles to form long 1D structures (wormlike micelles) at high pH at the concentrations discussed here.

Multicomponent systems can be used to gain extra control over supramolecular systems and provide easy access to new properties that are difficult to achieve from single component systems.^[^ ^1^, ^2^ ^]^ Multicomponent systems can conceptually form a range of different possible structures; they can self‐sort, where each self‐assembled structure formed only contains one of the components, or co‐assemble, where each structure formed contains a mixture of the components (**Figure** **1**a).^[^ ^3^, ^4^, ^5^ ^]^ Furthermore, co‐assembly can be orthogonal, cooperative, or destructive.^[^ ^6^ ^]^ During orthogonal co‐assembly, the components assemble independently in the presence of one another, forming distinct structural components.^[^ ^6^ ^]^ In cooperative co‐assembly, the structures formed contain an intimate mixture of each component.^[^ ^6^ ^]^ The arrangement of the components in such assemblies can be ordered, as is the case with donor‐acceptor systems,^[^ ^7^ ^]^ or may be random.^[^ ^8^ ^]^ Disruptive co‐assembly occurs where the co‐assembly of a mixture of components reduces the ability of the system to form self‐assembled structures.^[^ ^6^ ^]^ Each of these possibilities comes with potential for preparing new types of systems that cannot be accessed when using a single component.^[^ ^5^ ^]^

Co‐assembled systems are useful for many applications. For example, bioactive peptides can be incorporated into a multicomponent gel network to improve cell culture viability.^[^ ^9^ ^]^ Different modes of co‐assembly are suited for certain applications. Cooperative co‐assembly between structurally similar peptides can be used to achieve supramolecular charge transfer within stacked structures containing alternating donor and acceptor molecules for optoelectronic applications.^[^ ^10^ ^]^ Alternatively, self‐sorting of two components can be exploited to form interpenetrating networks of bulk heterojunctions containing domains of each individual component. Such architecture is desirable for p–n heterojunction photovoltaics.^[^ ^11^ ^]^

**Figure 2:**
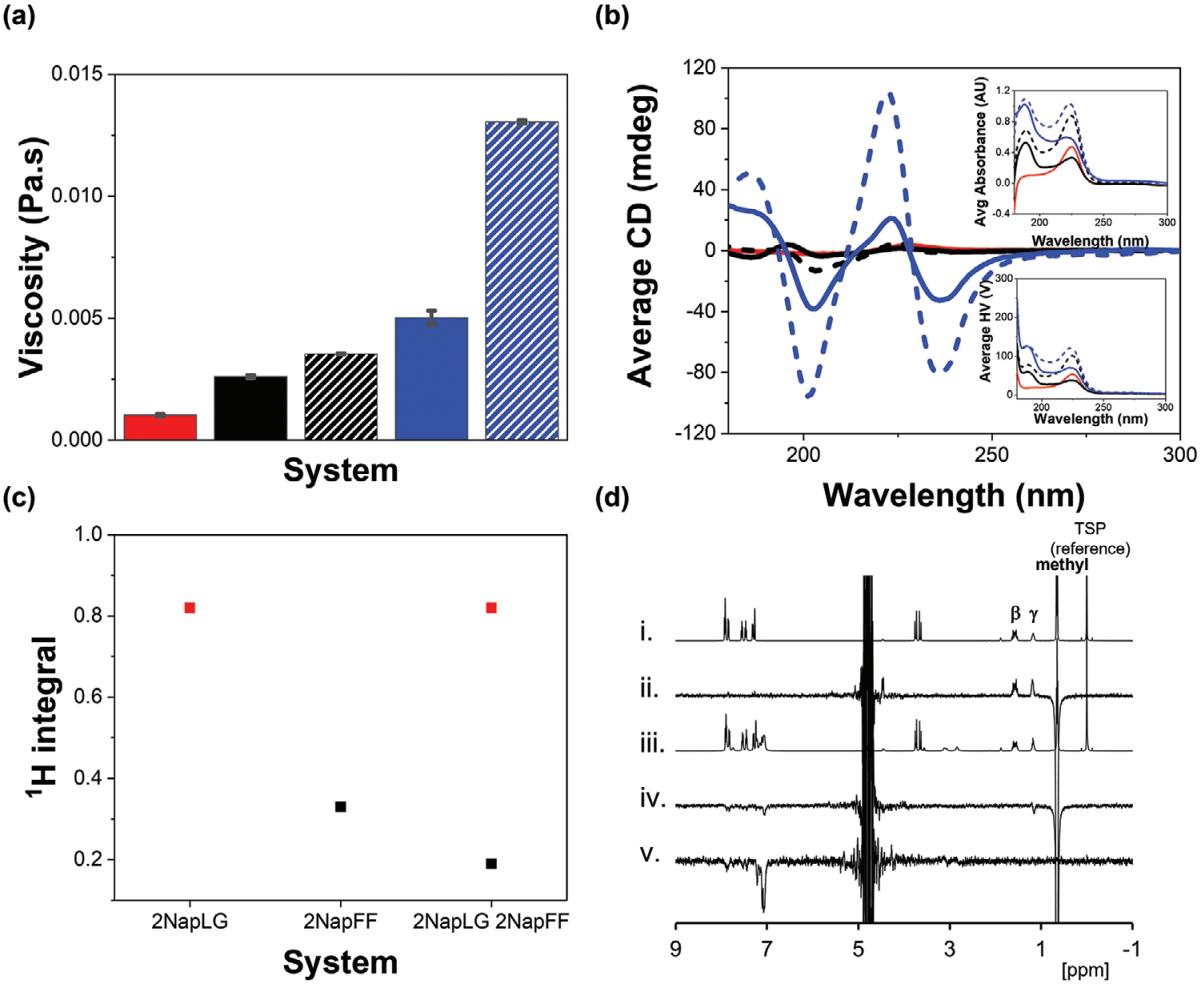
a) Average viscosity values recorded at a shear rate of 10 s^−1^ and b) CD spectra recorded for each system in H_2_O at pH 10.5; 2NapLG 2.5 mg mL^−1^ (red), 2NapFF 2.5 mg mL^−1^ (black), 2NapLG 2.5 mg mL^−1^:2NapFF 2.5 mg mL^−1^ (black dashed), 2NapFF 5 mg mL^−1^ (blue) and 2NapLG 2.5 mg mL^−1^:2NapFF 5 mg mL^−1^ (blue dashed). Viscosity data were recorded at a temperature of 25 °C in duplicate with a fresh sample for each repeat. The error bars show the standard deviation between the samples. CD data were recorded in triplicate on the same sample and averaged. The inserts show the absorbance (top) and HV (bottom) spectra recorded concurrently with the CD spectra. c) ^1^H integrals recorded from 2NapLG 2.5 mg mL^−1^, 2NapFF 5 mg mL^−1^ and 2NapLG 2.5 mg mL^−1^:2NapFF 5 mg mL^−1^. The integrals from the methyl group on 2NapLG are shown in red and those from the CH_2_Ph group on 2NapFF are shown in black. Integrals were normalized to the resonance from the TSP internal reference. All NMR spectra were collected at 25 °C using H_2_O as a solvent. d) ^1^H NMR spectra of: i) 2NapLG 2.5 mg mL^−1^ ii) 2NapLG 2.5 mg mL^−1^ showing the NOE difference when methyl resonance inverted, iii) 2NapLG 2.5 mg mL^−1^:2NapFF 5 mg mL^−1^ and, iv,v) 2NapLG 2.5 mg mL^−1^:2NapFF 5 mg mL^−1^ showing the NOE difference when methyl resonance inverted (iv) and when phenylalanine aromatic resonance inverted (v).

Here, we describe multicomponent systems composed of the two structurally similar N‐functionalized dipeptides 2NapLG (Figure 1b) and 2NapFF (Figure 1c). N‐Functionalized peptides with a free carboxylic acid at the C‐terminus behave like surfactants in solution at high pH.^[^ ^3^, ^6^ ^]^ Surfactants have previously been used to control the self‐assembly behavior of peptides.^[^ ^1^, ^3^, ^6^, ^12^ ^]^ Each dipeptide studied here has the capability to control the assembly of the other via its surfactant‐like properties.

For two components to effectively co‐assemble, they must share a common mode of assembly.^[^ ^6^, ^13^ ^]^ There is the potential therefore for 2NapLG and 2NapFF to co‐assemble at high pH in their micellar states as they both contain a N‐terminal naphthalene ring and a C‐terminal carboxylic acid. The naphthalene ring can drive assembly through the formation of π–π interactions and the hydrophobic effect.^[^ ^14^ ^]^ In such functionalized dipeptides‐based systems, the carboxylic acid can be used to trigger gelation via a reduction in pH^[^ ^15^ ^]^ or by formation of salt bridges on addition of divalent cations.^[^ ^16^ ^]^ Since 2NapLG and 2NapFF have different amino acid residues in their peptide chain, if co‐assembly were to occur, we would expect 2NapLG and 2NapFF to co‐assemble in an orthogonal manner (Figure 1aiii). Co‐assembly of such a system provides an avenue for tuning the properties of micellar solutions and any resulting gel phases.

## 2 Results and Discussion

Despite their structural similarities, 2NapLG and 2NapFF have very different behavior in aqueous solutions. 2NapFF forms wormlike micelles in solution at high pH.^[^ ^17^ ^]^ Such solutions have a viscosity greater than that of water and exhibit shear‐thinning behavior (Figure S3, Supporting Information).^[^ ^18^ ^]^ In comparison, 2NapLG forms non‐viscous solutions at high pH, suggesting that non‐persistent structures are formed by 2NapLG in solution at high pH.^[^ ^19^ ^]^ Solutions containing a mixture of 2NapFF and 2NapLG at concentration ratios of 2.5 mg mL^−1^:2.5 mg mL^−1^ and 2.5 mg mL^−1^:5 mg mL^−1^ (2NapLG:2NapFF) show greater viscosity than either component alone at the same concentrations at pH 10.5 (**Figure** **2**a). This is particularly interesting since 2NapLG alone shows no shear‐thinning behavior (Figure S3, Supporting Information) and is considerably less viscous than 2NapFF (Figure 2a). These data therefore strongly imply that the mixture of 2NapFF and 2NapLG are not operating in a self‐sorting fashion (where simple dilution of the 2NapFF would be expected to lead to a significant decrease in viscosity).

The integrals corresponding to protons on 2NapFF measured by ^1^H NMR spectroscopy are smaller in the presence of 2NapLG (Figure 2c), showing that more 2NapFF molecules are assembled in the multicomponent system.^[^ ^20^ ^]^ This agrees with the increased circular dichroism (CD) signals observed both as 2NapFF concentration increases and as 2NapLG is added to the system (Figure 2b). CD data on heating a solution of 2NapFF and a solution of 2NapFF mixed with 2NapLG show that the signal intensities persist to higher temperatures in the mixture as compared to the single component system (Figure S5). All these data show that 2NapLG is influencing the self‐assembly behavior of 2NapFF. The integrals corresponding to protons on 2NapLG remain the same in the presence and absence of 2NapFF (Figure 2c). Therefore, the mobility of 2NapLG molecules on an NMR timescale does not change going from the single component to a multicomponent system.

We confirmed that 2NapLG forms physical interactions with the structures formed by 2NapFF in solution at high pH using nuclear Overhauser effect (NOE) NMR experiments. In samples of 2NapLG alone, we observed a positive NOE difference to the β and γ protons when the methyl resonance was selectively inverted (Figure 2d). This confirms that 2NapLG behaves like a small molecule in solution at high pH, i.e., it does not form persistent micellar structures.^[^ ^21^ ^]^ However, in the presence of 2NapFF at 25 °C, a negative NOE difference was observed between the γ proton and the aromatic protons of 2NapFF (Figure 2div). These observations show that the 2NapLG is interacting with the large structures formed by 2NapFF.^[^ ^21^ ^]^ For 2NapFF, when the aromatic signal is selectively inverted, we observe NOE to other aromatic protons and to the CH_2_Ph groups of 2NapFF. The NOE difference is negative, as we would expect from the formation of worm‐like micelles (Figure 2dv).^[^ ^22^ ^]^

**Figure 3:**
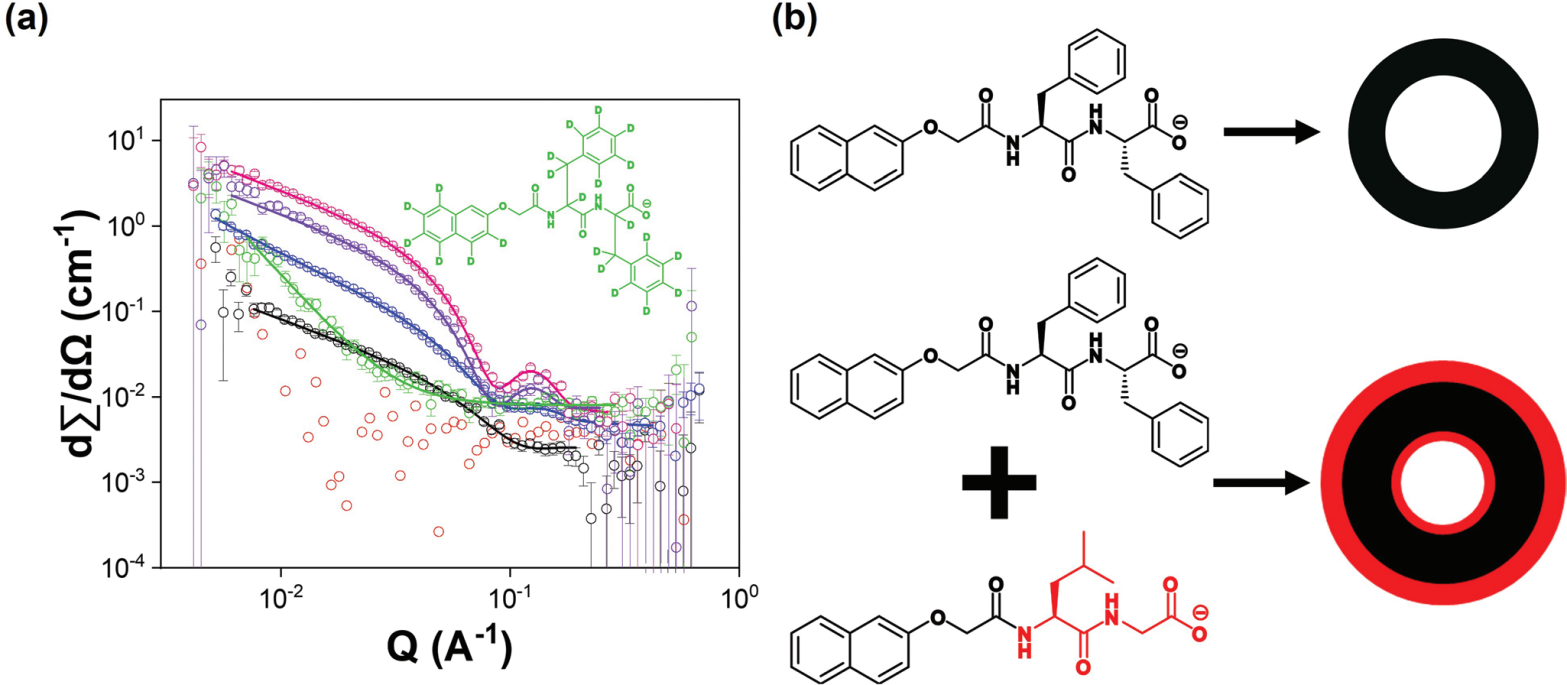
a) Plots of SANS data (circles) from 2NapLG 2.5 mg mL^−1^ (red), 2NapFF 2.5 mg mL^−1^ (black), 2NapLG 2.5 mg mL^−1^:2NapFF 2.5 mg mL^−1^ (blue), 2NapFF 5 mg mL^−1^ (pink), 2NapLG 2.5 mg mL^−1^:2NapFF 5 mg mL^−1^ (purple) and 2NapLG 2.5 mg mL^−1^:d2Nap‐dd‐FF 5 mg mL^−1^ (green). The fits are shown as solid lines. b) Schematic representation of how the systems studied here self‐assemble, looking down the length of the hollow cylinders formed.

SANS data provide further evidence that 2NapLG is interacting with the structures formed by 2NapFF at high pH. 2NapLG (2.5 mg mL^−1^ in D_2_O) alone does not scatter well enough to produce data of suitable quality for fitting (**Figure** **3**a). This agrees with viscosity and NMR data and confirms that 2NapLG alone does not form persistent micellar structures in an aqueous solution. 2NapFF alone forms long cylindrical structures in solution, commonly referred to as wormlike micelles. At a concentration of 2.5 mg mL^−1^, data collected from a sample of 2NapFF in D_2_O were fitted to a cylinder model with a radius of 28 Å. A summary of all the parameters obtained from fitting SANS data can be found in the Table S1 (Supporting Information). D_2_O was used as a solvent instead of H_2_O to provide sufficient contrast between the solvent and the structures for high‐quality data to be collected.^[^ ^23^ ^]^ Viscosity and CD experiments were repeated in D_2_O to confirm that the same behavior observed in H_2_O is also observed in D_2_O (Figures S3 and S4, Supporting Information).

SANS data collected from the 2NapLG 2.5 mg mL^−1^:2NapFF 2.5 mg mL^−1^ multicomponent system were fitted to a hollow cylinder model (Figure 3). The hollow cylinder had a radius of 13 Å and a thickness of 22 Å. This shows that co‐assembly with 2NapLG changes the structures formed by 2NapFF as the structures begin to resemble those formed at higher 2NapFF concentrations (Table S1, Supporting Information), despite 2NapLG alone not forming cylindrical structures.

When the concentration of 2NapFF was increased to 5 mg mL^−1^, the scattering data were fitted to a hollow cylinder model with a radius of 15 Å and thickness of 22 Å. The SANS data from the 2NapLG 2.5 mg mL^−1^:2NapFF 5 mg mL^−1^ multicomponent system were also fitted to a hollow cylinder model. The presence of 2NapLG resulted in changes to the structures as compared to those formed from 2NapFF alone. The radius of the inner core of the hollow cylinder decreased to 12 Å and the thickness of the outer tube increased to 28 Å. From comparison with similar work reported by Abul‐Haija et al.,^[^ ^1^ ^]^ we suggest that 2NapLG is behaving like a surfactant and is coating the inner and outer surfaces of the hollow cylinders formed by 2NapFF (Figure 3b). Co‐assembly of gelator and surfactant molecules results in the formation of functionalized nanofibers, with the functional groups exposed on the surfaces of the fibers.^[^ ^6^ ^]^ Despite partial incorporation of the surfactant into the self‐assembled structures, Abul‐Haija et al. reported that the core structure formed by the peptide‐based gelator was unaffected by the presence of the surfactant‐like component. Such systems can be thought of as undergoing orthogonal co‐assembly.

Selective deuteration of 2NapFF allowed us to probe how 2NapLG alone scatters in the multicomponent system.^[^ ^23^ ^]^ The mostly deuterated analog, d2Nap‐dd‐FF (Figure 3), forms viscous solutions like 2NapFF but does not scatter well by SANS as expected due to the lack of contrast. Interestingly, samples composed of 2NapLG:d2Nap‐dd‐FF at either concentration ratio also do not scatter well. The loss of scattering intensity on removal of the scattering contribution from 2NapFF suggests that 2NapLG is forming weakly scattering shells on the inner and outer surfaces of the structures formed by 2NapFF.

2NapFF can form gels at high pH on addition of calcium ions.^[^ ^16^ ^]^ The divalent cations form cross‐links between deprotonated carboxylic acid groups on neighboring worm‐like micelles, resulting in formation of a network.^[^ ^16^ ^]^ The 2NapLG 2.5 mg mL^−1^:2NapFF 5 mg mL^−1^ multicomponent system also formed stable gels on addition of a calcium salt (Figure S6, Supporting Information). The 2NapLG 2.5 mg mL^−1^:2NapFF 5 mg mL^−1^ multicomponent gels had similar stiffness (*G*′) to the 2NapFF (5 mg mL^−1^) single component gels but had a much higher critical strain (strain value at which G″ crosses over *G*′), signifying greater strength (Figure S7, Supporting Information).

Interest is growing in supramolecular noodles, first reported by Zhang et al.,^[^ ^24^ ^]^ for optoelectronics^[^ ^25^ ^]^ and regenerative medicine^[^ ^26^ ^]^ and as tough, flexible materials.^[^ ^27^ ^]^ The ability of the 2NapLG 2.5 mg mL^−1^:2NapFF 5 mg mL^−1^ multicomponent system to form gels on addition of CaCl_2_ was a good indication that this system would form supramolecular noodles. The higher viscosity in the solution phase and greater mechanical strength in the gel state of the multicomponent system led us to hypothesize that the inclusion of 2NapLG would allow formation of more robust supramolecular noodles than those formed by 2NapFF alone.

**Figure 4:**
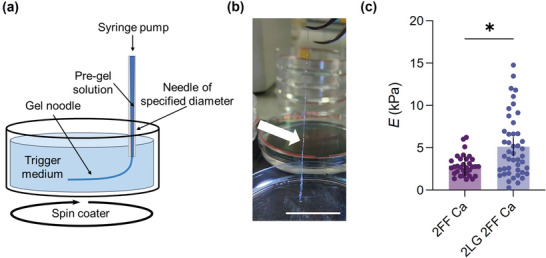
a) Schematic showing how the noodles were prepared using the syringe pump and spin coater setup. CaCl_2_ (0.5 m, aqueous solution) was used as the trigger medium by providing Ca^2+^ ions which cross‐link the gelator molecules, allowing formation of a gel network. b) Photograph of 2NapLG 2.5 mg mL^−1^:2NapFF 5 mg mL^−1^ supramolecular noodle during transfer from its trigger medium. Scale bar: 3 cm. c) Young's modulus (*E*) of noodles composed of 2NapFF 5 mg mL^−1^ (2FF Ca) and 2NapLG 2.5 mg mL^−1^:2NapFF 5 mg mL^−1^ (2LG 2FF Ca). Mean ± 95% CI, *n* = 33 for 2FF Ca, *n* = 48 for 2LG 2FF Ca obtained from at least two separate noodles, *p < 0.05, two‐tailed Mann–Whitney test.

Noodles were prepared using the syringe pump and spin‐coater setup as described elsewhere (**Figure** **4**a).^[^ ^28^ ^]^ The noodles formed were sufficiently robust to be transferred from the plastic petri dish in which they were formed to a glass surface to allow nanoindentation measurements to be performed (Figure 4b). However, it was clear that the 2NapFF noodles were less mechanically robust than the multicomponent noodles (Figure S8 Supporting Information). We recorded maps of indentations^[^ ^29^ ^]^ along the length of several noodles prepared from the 2NapFF 5 mg mL^−1^ single component system and from the 2NapLG 2.5 mg mL^−1^:2NapFF 5 mg mL^−1^ multicomponent system; and quantified the mechanical response by fitting force‐indentation data with the Hertz model to obtain Young's modulus (*E*), as explained in the experimental methods. Multicomponent noodles have greater *E* than single component noodles (Figure 4c), showing how the mechanical properties of such gel noodles can be modulated by the addition of a second component.

**Figure 5:**
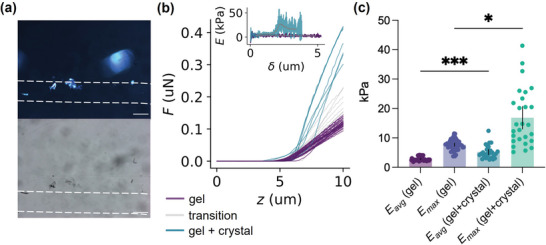
a) Optical microscopy images collected under cross‐polarized (top) and non‐polarized light (bottom). The white dashed lines highlight the outer edges of the noodles. Scale bars: 300 µm. b) Representative force (*F*)–distance (*z*) nanoindentation curves of a composite noodle transitioning from a region devoid of crystal (gel, purple) to a region containing a crystal (gel + crystal, green). The increased slope represents an increase in stiffness. The inset shows *E* as a function of indentation depth, δ, obtained using the elasticity spectra approach^[^ ^31^ ^]^ for a region devoid of crystal (purple) and for a region containing a crystal (green). The gray traces are smoothed data. c) Young's modulus (*E*) of composite noodles containing crystals obtained using the elasticity spectra approach. Data were collected from regions without crystals (gel) and from regions containing crystals (gel + crystal) and Young's modulus calculated as a function of the indentation depth. The bars show for each condition the value of the soft (*E*_avg_) and stiff components (*E*_max_) (see experimental section). Mean ± 95% CI, *n* = 51 for gel and *n* = 27 for gel+crystal obtained from at least 2 separate noodles, * *p* = 0.0337, *** *p* = 0.0005, One‐way ANOVA with Kruskal‐Wallis multiple comparison test.

Micellar dispersion of 2NapFF at high pH form supramolecular hydrogels on a reduction in pH using glucono‐δ‐lactone (GdL).^[^ ^15^ ^]^ 2NapLG forms crystals under the same conditions (Figure S9, Supporting Information). By slightly reducing the pH of the environment surrounding pre‐formed 2NapLG:2NapFF gel noodles to just below that of the first apparent p*K*_a_ of the system (Figure S11, Supporting Information), we were able to form crystals within the gel noodles (**Figures** **5**a; Figure S10, Supporting Information). The formation of crystals within the gel noodles shows that the system is transitioning from co‐assembly to self‐sorted assembly when the pH is reduced below a critical value. Crystals were able to form at pH values as high as 8.0 by simply transferring the noodles from the CaCl_2_ solution in which they were formed to a bath of deionized H_2_O (pH 7–8). We expect that the same effect as observed in similar multicomponent systems is taking place, where charge is removed from the component with the higher apparent p*K*_a_ first.^[^ ^30^ ^]^ Such charge removal drives self‐assembly of the first component, while the second component still carries its charge and thereby remains dispersed in solution. The pH change on transfer from CaCl_2_ solution deionized H_2_O allows the system to spend sufficient time at a pH value that is below the apparent p*K*_a_ of the first component (in this case 2NapFF, Figure S11, Supporting Information) and above the apparent p*K*_a_ of the second (2NapLG, Figure S11, Supporting Information), resulting in a preference for self‐sorting.

The kinetics of pH reduction, therefore, plays an important role in crystal formation. For example, no crystal formation was observed when the multicomponent noodles were transferred directly into an acidic solution, e.g., 0.1 m HCl. Such fast pH reduction does not allow sufficient time at the appropriate pH value for self‐sorting to occur. It is possible that secondary nucleation occurs in the co‐assembled structure, but this is a difficult aspect to probe.

While no polymorphism was observed between the different 2NapLG crystals formed in the presence and absence of 2NapFF at low (pH ≈ 3.5), 2NapLG crystals formed at higher pH (pH ≈ 10.0) on addition of CaCl_2_ had different morphology (Figure S9, Supporting Information) and PXRD pattern (Figure S14, Supporting Information) to those formed at low pH.

Nanoindentation was again used to investigate the effect of crystal formation on the mechanical properties of the noodles. Figure 5b clearly shows a marked increase in the slope of the indentation curves (apparent stiffness)^[^ ^32^ ^]^ when transitioning from a region with the gel only to an area where crystals are present. We were unable to perform nanoindentation measurements on the single component noodles following a reduction in pH as reducing the pH causes the noodles to become more brittle and break during transfer to the surface requiring form indentations to be performed.

Because of the non‐homogeneous nature of the sample, we calculated the elasticity spectra,^[^ ^31^ ^]^ computing *E* as a function of indentation depth, δ^[^ ^31^ ^]^ for regions containing the gel only and the gel plus crystal (Figure 5b, inset). To obtain quantifiable metrics of the elasticity of the system, we computed the soft (*E*_avg_) and hard (*E*_max_) components of each elasticity spectrum (see experimental section). As observed in Figure 5c, the crystals impact on the mechanical properties of both phases, and, as expected, the increase in the rigid component is much more pronounced.

We were able to achieve the same outcomes using a similar second component to 2NapLG, PhOLL (PhOLL as a second example, Supporting Information), thus showing that this is not an exceptional case. We can therefore use this multicomponent system approach to prepare new composite materials.

## 3 Conclusions

We have shown how the preparation of multicomponent systems from two structurally similar, but behaviorally different components can be used to modulate the properties of a low‐molecular‐weight gelator in both the sol and gel phases. Using this multicomponent system, we have exemplified the potential of multicomponent supramolecular noodles and how the interesting behavior of the individual components can be exploited to achieve new behaviors. We can harness the crystal‐forming behavior of one component and the supramolecular gel noodle‐forming behavior of a second component to achieve a composite material with both solid and viscoelastic behavior. These unusual materials significantly expand the scope of such soft materials.

## Conflict of Interest

The authors declare no conflict of interest.

## Supporting information

Supporting Information

## Data Availability

The data that support the findings of this study are available from the corresponding author upon reasonable request.
