# Supplementary material for: Hierarchical Composite Self‐Sorted Supramolecular Gel Noodles
Source: Adv Mater. 2023 Mar 15;35(17):2211277. doi: 10.1002/adma.202211277 (PMC11475401; doi:10.1002/adma.202211277)
Supplement: Supplementary file 1 — Supporting Information [file ADMA-35-2211277-s001.pdf]

ADVANCED MATERIALS

Supporting Information

for *Adv. Mater.*, DOI: 10.1002/adma.202211277

Hierarchical Composite Self-Sorted Supramolecular Gel Noodles

*Libby J. Marshall, Matthew Wallace, Najet Mahmoudi, Giuseppe Ciccone, Claire Wilson, Massimo Vassalli, and Dave J. Adams**

Supporting Information

The supporting information (ESI) contains experimental methodology; full viscosity data; viscosity, CD, NMR and rheology data discussed but not presented in the main text; summaries of all the parameters obtained from fitting SANS data; photographs of gels and crystals; PXRD data; PhOLL as a second example and characterization of the materials synthesized for this work.

Hierarchical Composite Self-Sorted Gel Noodles

*Libby J. Marshall,[a] Matthew Wallace,[b] Najet Mahmoudi[c], Giuseppe Ciccone[d], Claire Wilson,[a] Massimo Vassalli[d] and Dave J. Adams[a],**

Experimental Methods

Materials

All chemicals were purchased from Sigma-Aldrich and used as received. Deionized water or 99.9 % D_2O from Sigma Aldrich was used throughout. 2NapFF and 2NapLG were synthesized as previously described.[33]

Preparation of micellar solutions

Solutions were prepared by suspending the gelators separately in water with the addition of 1 molar equivalent of NaOH (0.1 M). The suspensions were stirred overnight to ensure complete dispersion of the molecules. The multicomponent solutions were prepared by first preparing separate stock solutions of the two components at twice the desired concentration before adjusting the pH to 10.5 by addition of 1 M NaOH and mixing the stock solutions together in a 1:1 ratio. NaOH of a higher concentration was used for pH adjustment to reduce the volume being added to the solutions and thereby minimize any changes to the concentrations of the solutions. The solutions were adjusted to pH 10.5 again before analysis.

pH measurements and pK_a Titrations

The pH of samples was measured using a FC200 pH probe from Hanna instruments. The pH meter was calibrated using pH 4.01, 7.01 and 10.01 buffer solutions. The probe was rinsed with deionised water between measurements. The pK_a values of 2NapLG (2.5 mg/mL), 2NapFF (5 mg/mL) and the multicomponent system 2NapLG 2.5 mg/mL:2NapFF 5 mg/mL were determined by titration *via* addition of aliquots of 0.1 M HCl solution.

Viscosity

Viscosity measurements were performed on an Anton Paar Physica 301 rheometer using a CP50 cone and plate (sample volume ~1 mL). Viscosity values were collected at shear rates ranging from 1-100 or 1-1000 s^{-1}. Measurements were performed in each condition in duplicate or triplicate and the average calculated. The solutions were transferred onto the plate by pouring. The temperature of the plate was set to 25 °C for the 25 °C samples. For each of the 10 °C samples, the sample was loaded onto the plate at 25 °C. The plate was then cooled to 10 °C at a rate of 0.5 °C/s. The sample was allowed to equilibrate at 10 °C for 5 minutes before the viscosity measurement was carried out with temperature maintained at 10 °C. During the heat-cool cycles, the temperature of the plate was increased or decreased at a rate of 0.5 °C/s. The sample was then held at the temperature at which the next measurement was to be taken for 5 minutes to allow the sample to equilibrate to the new temperature before the viscosity measurement was carried out.

Strain, Frequency and Time Sweeps

All rheological measurements were carried out using an Anton Paar Physica MCR 301 rheometer at 25 °C. Strain, frequency and time sweeps were performed using cup and vane geometry. Strain sweeps were performed at a frequency of 10 rad/s from 0.01% to 1000% strain. Frequency sweeps were performed at 0.1% strain from 1 rad/s to 100 rad/s frequency. Time sweeps were performed at 0.5% strain and 10 rad/s frequency. Samples were prepared with 2 mL volume in 7 mL Sterilin vials.

Circular Dichroism

Circular dichroism (CD) was measured using a Chirascan VX CD spectrometer (Applied Photophysic Limited, U.K.) using a quartz cell with a 0.01 mm path length and the following parameters: scanning mode, continuous; scanning speed, 120 nm/min and bandwidth, 1 nm. All CD data are presented as ellipticity and recorded in millidegree (mdeg). Absorbance spectra were recorded concomitantly with CD spectra. Spectra were obtained at a 2 min interval from 180 to 300 nm at a speed of 20 nm/min. All spectra were recorded in triplicate and averaged with the exception of the temperature-variable CD experiment. These were recorded as a single measurement on the same sample at each temperature. CD spectra were recorded at intervals of 5 °C at temperatures ranging from 25 °C to 95 °C (the upper limit of the CD spectrometer).

NMR

Nuclear Overhauser (NOE) experiments and ^{1}H experiments (Figure 2) were performed on a Bruker 500 MHz spectrometer equipped with a Neo console and Bruker 5 mm SmartProbe™. Solutions of the gelators were prepared in H_2O, as described above, and transferred to Wilmad

5 mm diameter 528-PP NMR tubes. A separate 2.0 mm outer diameter capillary (Bruker) containing 30 mM 3-(trimethylsilyl)propionic-2,2,3,3-d_4 acid sodium salt (TSP) in D_2O was inserted into the sample in the NMR tube to provide a chemical shift and integral reference (0 ppm). ^{1}H experiments were recorded using the perfect echo WATERGATE sequence of Adams et al.[34] incorporating the double echo W5 sequence of Liu et al.[35] The delay between successive pulses in the selective pulse train was set at 333 μs, corresponding to 3000 Hz between the null points. Spectra were acquired in 128 scans with a relaxation delay of 5 s and signal acquisition time of 4.2 s. 1D selective NOE experiments were acquired using the Bruker library sequence, selnogp.[36] An 80 ms Gaussian pulse was used to selectively invert the methyl resonance of 2NapLG. Spectra were acquired in 128 scans with a relaxation delay of 2 s and signal acquisition time of 3.3 s. ^{1}H integrals were obtained relative to TSP internal reference.

SANS

Small-angle neutron scattering (SANS) measurements were performed on the SANS2D instrument at the ISIS Neutron and Muon source (STFC Rutherford Appleton Laboratory, Didcot, UK), using an Anton Paar Physica MCR 502 rheometer equipped with a bespoke titanium Couette geometry and a temperature controller (TC-30). The Couette cell has a gap of 0.5 mm and was set up for the neutron beam to shine in the radial direction. The incident neutron beam was 8 mm in diameter with a wavelength range of 1.75-16.5 Å and a sample-to-detector distance of 4 m, resulting in an effective range of scattering wave vector, q, of 0.004-0.7 Å^{-1}:

$$q = \frac{4\pi}{\lambda} sin\left({}^{\theta}/_{2}\right) \; (1),$$

where λ is the neutron wavelength and θ is the scattering angle. Raw scattering data are corrected for sample transmission, detector efficiency and solvent scattering[37] using the Mantid software, and then converted to absolute scattering cross section (I(q)/cm^{-1}) using the scattering from a standard sample (a solid blend of hydrogenous and perdeuterated polystyrene) in line with established procedures.[38] Data modelling was performed using the Sasview package.[39]

Due to shear alignment in the y-plane resulting from loading of the samples into the rheometer, certain samples underwent additional processing prior to fitting. This additional processing was only performed on samples that exhibited shear artefacts (see Figure S1 for example). The scattering from -45 to 45° was cut from the 2D scattering pattern and only the scattering from 45 to 135° was used for data modelling. Such analysis has been previously referred to as bow-tie integration.[28]

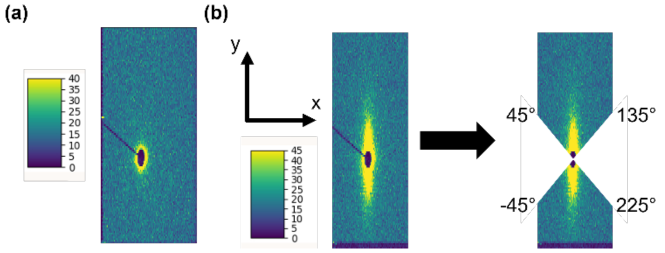

Figure S1. (a) Example of 2D scattering pattern from d-2Nap-dd-FF which did not show any shear alignment and therefore was not processed using bowtie method. (b) Example of 2D scattering data from 2NapFF showing alignment along the *y*-axis, resulting in low intensity scattering in the x-direction. All data sets that exhibited shear alignment were subject to vertical bowtie integration where the weak scattering from between -45° and 45° (and between 135° and 225°) was cut from the 2D scattering pattern (as shown in Figure 1b) and only scattering from between 45° and 135° (and between 225° and -45°) was used in model fitting. This data was of better quality than the averaged data.

Preparation of Ca-triggered gels

Gelator solutions were prepared as previously described. 2 mL of solution adjusted to pH 10.5 was transferred to a 7 mL Sterilin vial. 2 molar equivalents (according to the total mass of peptide) of $CaCl_2$ (200 mg/mL aqueous solution) were pipetted on top of the centre of solution in the Sterilin vial. The samples were left to rest for 3 days at room temperature to allow formation of stable and reproducible gels before rheological measurements were performed.

Preparation of supramolecular noodles

Noodles could be prepared by extrusion from a pipette directly into a static $CaCl_2$ solution (0.5 M). Longer, more homogenous noodles were formed using a combined syringe pump-spin coater method described in other work from our group: an Alaris Carefusion syringe pump was used to control the flow rate of the peptide solutions. A 10 mL syringe was attached via a Luer lock fitting to 20 cm rubber tubing with a flat-headed needle (413 μm inner diameter) at the end. To load the syringe pump, the syringe was filled directly with gelator solution. The tubing (already connected to the needle) was attached to the syringe and the pre-gel solution pumped manually until the tubing was full. The tubing and needle were held vertically to prevent the formation of air bubbles. The syringe was then loaded into the syringe pump. The rubber tubing enabled movement of the needle to allow positioning into the trigger medium ($CaCl_2$, 0.5 M aqueous solution). The flow rate on the syringe pump was set to 100 mL/hr. The spin-coater was set to spin at a rate of 100 rpm. A plastic petri dish (900 mm diameter) containing $CaCl_2$ solution (0.5 M, 20 mL) was placed on the center of the spin coater plate. The needle connected to the syringe pump was held vertically into the petri dish. The length of the noodles could be

altered by changing the length of time the needle was held in the $CaCl_2$ solution: longer time equates to longer noodles. Noodles prepared by this method were used for nanoindentation to increase homogeneity and thereby increase reproducibility of the mechanical properties along the length of the noodles.

Preparation of composite gel noodles

Composite gel noodles were prepared by first preparing noodles from the multicomponent 2NapLG 2.5 mg/mL:2NapFF 5 mg/mL system as described above. The noodles were then transferred to a petri dish of deionised water and left for 3 days to allow sufficiently large and numerous crystals to form. pH 7-8 was sufficient to consistently observe formation of crystals without compromising the strength of the noodles. Faster and further reduction in pH using 0.1 M HCl or GdL solutions resulted in formation of lower quality and fewer, if any, crystals and caused the noodles to become too brittle to be transferred onto a glass surface for nanoindentation.

Quasi-static nanoindentation experiments

Nanoindentation measurements were carried out using a nanoindentation device (Chiaro, Optics11, Amsterdam, NL) mounted on top of an inverted microscope (Zeizz Axiovert 200M) equipped with two linear polariser films (Thorlabs, Newton, New Jersey, USA) in cross configuration. Measurements were performed following the standardised protocol described by Ciccone et al.[29] using a cantilever with stiffness (k) of 0.5 Nm^{-1} holding a spherical tip of radius *(R)* of 3 μm.

Each noodle was placed in a petri dish and aligned along the x direction of the microscope stage and stabilised using metal washers placed at either end on the sample. All measurements were carried out at room temperature (~ 23 °C) in milliQ water to maintain samples' hydration. For each experimental condition, at least 2 noodles were tested by performing a minimum of 2 matrix scans on each sample along its length. Each matrix scan consisted of ~ 20 indentations with spacing between subsequent indentations of 10 μm, or smaller when indentations were performed over a crystal due to its small size. For each indentation the probe moved at a speed of 2 μms^{-1} over a vertical range of 10 μm.

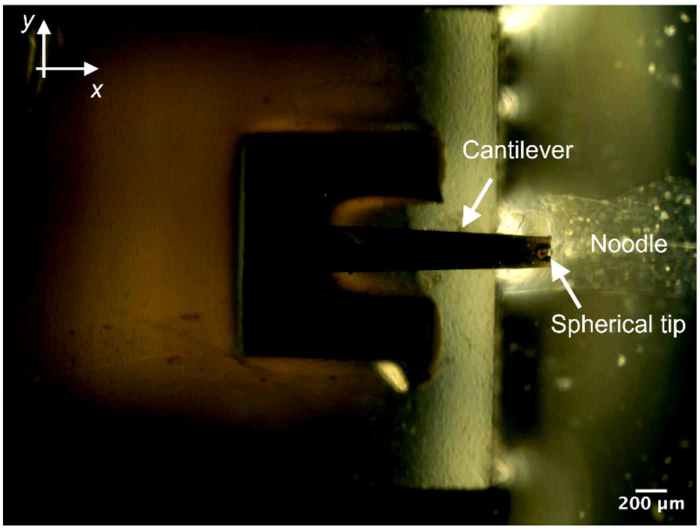

Figure S2. Photograph of gel noodle under cross-polarised light with indenter probe. Axes show direction of probe movement.

The forward segment of the collected force-displacement (*F*-*z*) curves was analysed using a custom open-source software.[29] Briefly, curves were first filtered using a Savitzky Golay filter from the SciPy computing stack[40] with window length of 25nm and polynomial order of 3. After, the point where the probe came into contact with the sample (z_0, F_0) was identified with the ratio of variance (RoV) algorithm or a thresholding algorithm[41] to convert *F*-*z* curves into force-indentation (F-δ) curves. To quantify the elastic properties of the gels, F-δ curves were fitted with the Hertz model (Equation 1) up to a maximum indentation of $\delta = 0.1\,R$. The Poisson's ratio (ν) was taken as 0.5 assuming material's incompressibility given the hydrated nature of the samples.

$$F = \frac{4}{3}\frac{E}{(1-\nu^2)}\delta^{\frac{3}{2}}R^{\frac{1}{2}} \text{ (2).}$$

Alternatively, the elasticity spectra of the data were computed:[29, 31]

$$E(\delta) = \frac{3}{8\sqrt{R}}\frac{1}{\delta}\frac{dF}{d\delta} \text{ (3),}$$

To quantify the difference between conditions, we extracted two values from each spectrum, E_{avg} and E_{max}, corresponding to the soft and hard component of the spectrum, respectively. The soft component E_{avg} was calculated as the average elasticity in a depth range between 2000 nm and 3000 nm of indentation, where crystals were never found. Instead, the hard component E_{max} was calculated as the average of the values of the elasticity spectrum lying above the 90th percentile.

PXRD

PXRD (powder X-ray diffraction) patterns were collected using a Rigaku MiniFlex 6G equipped with a D/teX Ultra detector, a 6-position (ASC-6) sample changer and Cu sealed tube ($K\alpha1$ and $K\alpha2$ wavelengths - 1.5406 and 1.5444 Å respectively). Patterns were measured as $\theta/2\theta$ scans typically over a range of $3>2\theta>60°$. Data collection and analysis were carried out using Rigaku SmartLab Studio II software (Rigaku Corporation, 2014).

Optical Microscopy

Optical microscope images were taken of samples with and without magnetic field using a Nikon Eclipse LV100 microscope at 5x magnification. Images were collected under cross-polarised and non-polarised light. Scale bars were added to images using the software, ImageJ.

Statistical Analysis

Viscosity, rheology and CD spectroscopy data were collected in duplicate or triplicate, as stated in the experimental section. Origin 2020 graphing & analysis software was used to calculate the mean and standard deviation for each data set.

Statistical analysis was performed on data obtained from nanoindentation experiments (Figure 4, Figure 5, Figure S21). For each experimental condition, at least two separate noodles were indented, and successive indentations performed along the length of the sample (the specific number of indentations, n, is given in each figure caption; n> 20 for all experimental conditions). Data is reported as mean $\pm$ 95% CI.

Normality was evaluated by a D'Agostino & Pearson test, and the appropriate statistical test was then performed to evaluate statistical significance (significance level set at 0.05) as described in each figure caption. No outliers were removed.

All statistical analysis was performed in GraphPad Prism 9.

Supplementary Data

Viscosity Data

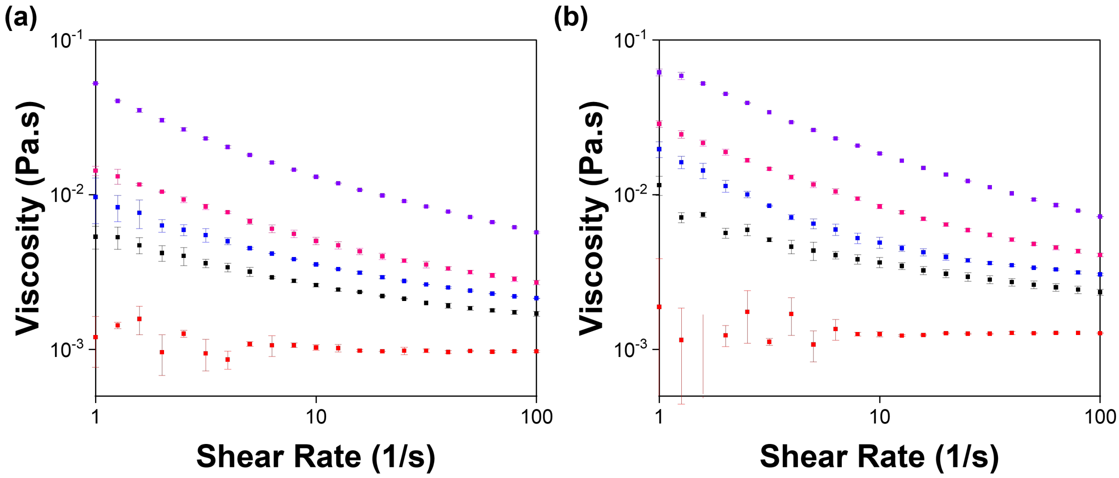

Figure S3. Full range viscosity data from the following samples: 2NapLG alone 2.5 mg/mL (red), 2NapFF alone 2.5 mg/mL (black), 2NapLG 2.5 mg/mL:2NapFF 2.5 mg/mL (blue), 2NapFF alone 5 mg/mL (pink) and 2NapLG 2.5 mg/mL:2NapFF 5 mg/mL (purple). Data was collected at 25 °C in (a) H_2O at pH 10.5 and (b) D_2O at pD 10.5. Data was collected in duplicate with a fresh sample for each repeat. The error bars show the standard deviation between the samples.

CD data

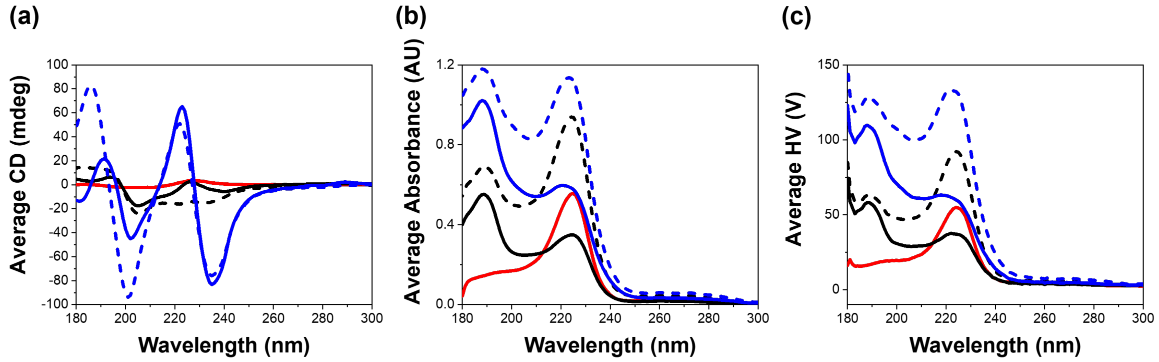

Figure S4. (a) CD spectra, (b) absorbance and (c) HV spectra collected from 2NapLG alone 2.5 mg/mL (red), 2NapFF alone 2.5 mg/mL (black), 2NapLG 2.5 mg/mL:2NapFF 2.5 mg/mL (black dashed), 2NapFF alone 5 mg/ml (blue), 2NapLG 2.5 mg/mL:2NapFF 5 mg/mL (blue dashed) in D_2O at pD 10.5. All data was collected in triplicate and averaged.

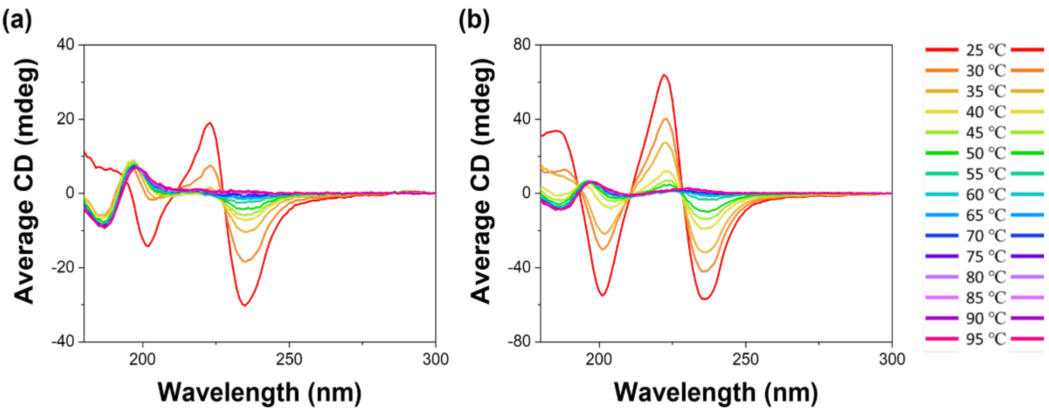

Figure S5. CD spectra recorded for (a) 2NapFF (5 mg/mL) and (b) 2NapLG (2.5 mg/mL):2NapFF (5 mg/mL). The temperature was increased in 5 °C increments between each recording, starting from 25 °C and ending at 95 °C (the upper limit of the CD spectrometer). The temperature at which each spectrum was recorded is shown in the key.

SANS data

Sample`	2FF 2.5 25C 0shear	2LG 2FF 2.5 25C 0shear	2FF 5 25C 0shear	2FF 5 25C 1000shear	2LG 2FF 5 25C 0shear	2LG 2FF 5 25C 1000shear
**Scale**	7.55 x10-5	1.85 x10-4	0.0018	0.0015	0.0007	0.0019
Scale error	*3.55 x10-6*	*2.71 x10-6*	*4.63 x10-5*	*3.73 x10-5*	*5.62 x10-5*	*4.75 x10-5*
**Background**	0.0024	0.044	0.0065	0.0080	0.0074	0.006
**Radius**	**28.16**	**13**	**15.119**	**14.82**	**12.06**	**14.78**
Radius error	*0.73*	-	*0.40*	*0.45*	*2.00*	*0.46*
Radius polydispersity	-	-	*0.25*	-	-	-
**Thickness**	**-**	**22**	**21.75**	**23.47**	**27.79**	**23.87**
Thickness error	-	-	*0.65*	*0.68*	*2.92*	*0.70*
**Length**	5000	5000	5000	5000	5000	5000
**Reduced chi2**	**1.13**	**4.38**	**2.12**	**4.49**	**1.58**	**2.85**

Table S1. Fitting parameters obtained from SasView model fitting of the SANS data. Parameter errors are fitting errors. 2NapFF 2.5 mg/mL (2FF 2.5) was best fit to a cylinder model. All other data sets were best fit to a hollow cylinder model; this model provided the best fit by eye and lowest chi2 values. Length was set to 5000 Å as the length of the structures under investigation is beyond the length scale measurable by SANS. The scattering length density (SLD) SLD, calculated using the NIST SLD calculator,[42] of 2NapFF (2FF) is 2.73 x 10^{-6} Å^{-2}; SLD of 1:1 2NapLG:2NapFF (2LG 2FF 2.5) is 2.5265 x 10^{-6} Å^{-2}; SLD of 1:2 2NapLG:2NapFF (2LG 2FF 5) is 2.594 x 10^{-6} Å^{-2} and SLD of D_2O (the solvent) is 6.393 x 10^{-6} Å^{-2}.

Ca^{2+} Gels

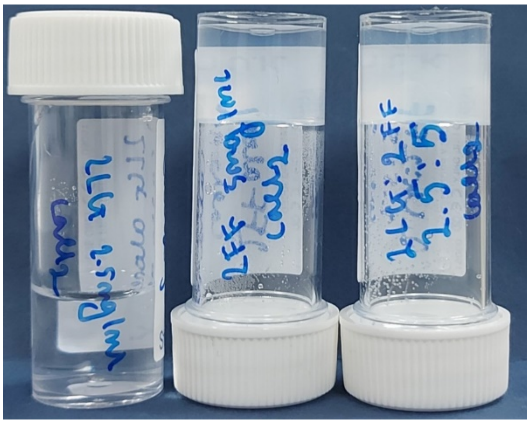

Figure S6: Left to right: 2NapLG alone 2.5 mg/mL, 2NapFF alone 5 mg/mL, 2NapLG 2.5 mg/mL: 2NapFF 5 mg/mL multicomponent system. Solutions of each system at pH 10.5 were "triggered" by addition of 2 molar equivalents of $CaCl_2$ (200 mg/mL aqueous solution) such that the final ratio of Ca^{2+} ions to functionalized-peptide molecules was 2:1. 2NapLG alone did not form a gel while 2NapFF alone and the 2NapLG/2NapFF mixture both formed gels.

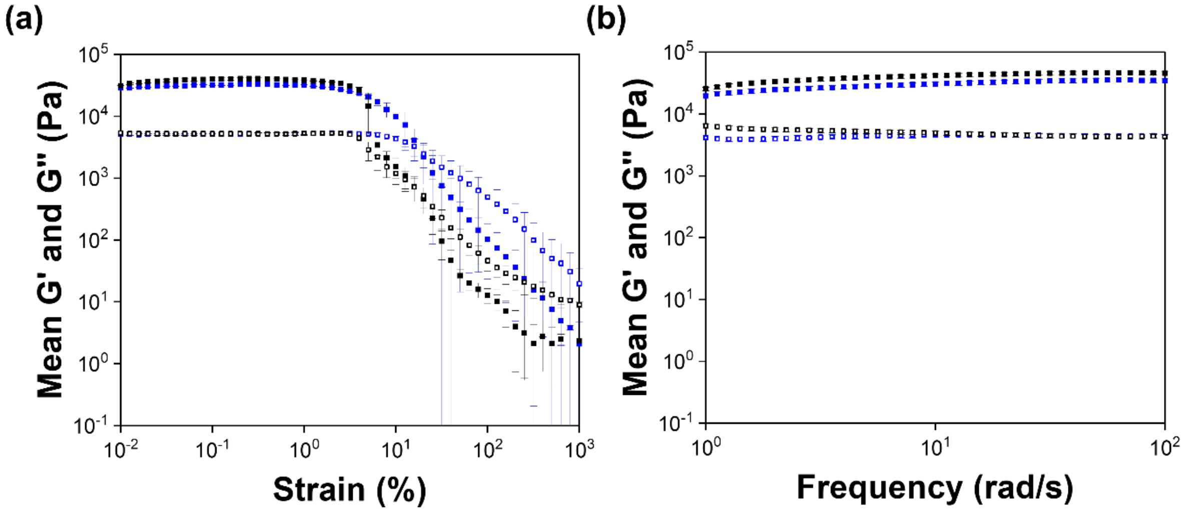

Figure S7: (a) Strain sweeps and (b) frequency sweeps of ca-triggered gels composed of 2NapLG 2.5 mg/mL:2NapFF 5 mg/mL (blue) and 2NapFF 5 mg/mL (black). Experiments were performed in triplicate with a fresh sample for each repeat. The error bars show the standard deviation between samples. The samples were allowed to rest for 3 days following addition of the Ca trigger to allow reproducible data to be collected.

The strain sweep shows that the 2NapFF alone and the 2NapLG/2NapFF gels have similar stiffness but that the multicomponent gels require application of significantly greater strain before complete breakdown of the gel network (where G′ crosses over with G″).

Noodles

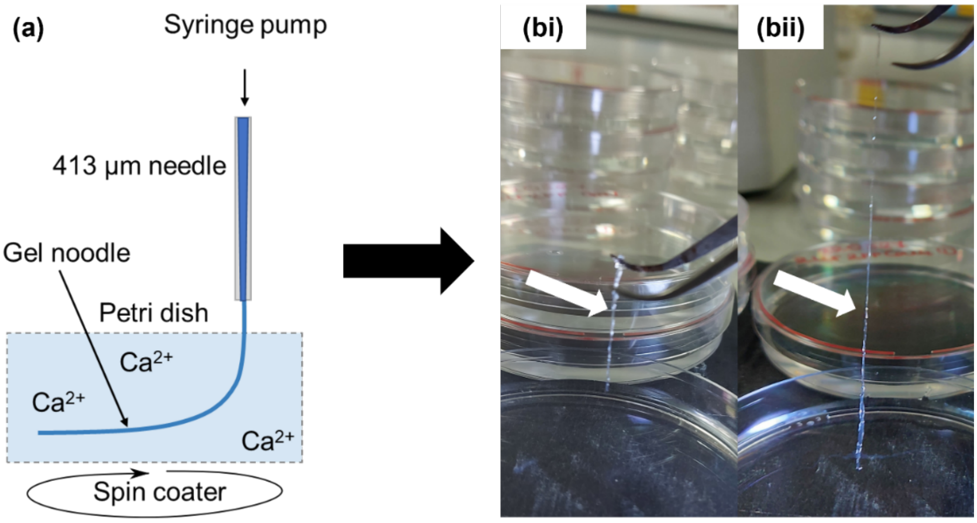

Figure S8. (a) Schematic showing how the noodles were prepared using the syringe pump and spin coater set up.[28] $CaCl_2$ (0.5 M, aqueous solution) was used as the trigger medium by providing Ca^{2+} ions which cross-link the gelator molecules, allowing formation of a gel network. (b) Photographs of (i) 2NapFF 5 mg/mL single competent and (ii) 2NapLG 2.5 mg/mL:2NapFF 5 mg/mL multicomponent noodles during transfer from the $CaCl_2$ solution in which they were formed to DI H_2O for crystal formation. The white arrows highlight the gel noodles.

Optical Microscopy of 2NapLG crystals

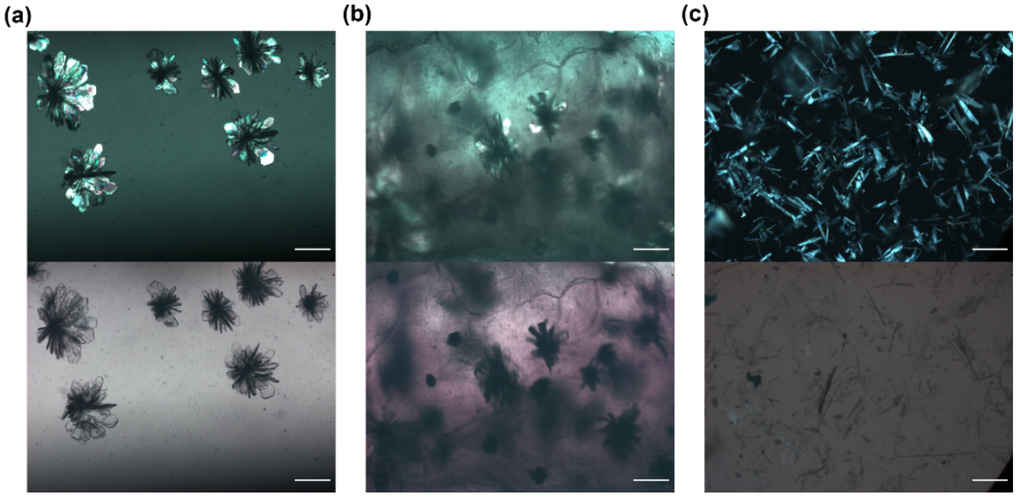

Figure S9. Crystals formed from aqueous solutions of (a) 2NapLG alone and (b) 2NapLG and 2NapFF formed by a reduction in pH using GdL as a trigger and (c) 2NapLG alone using $CaCl_2$ as a trigger. The starting pH of all solutions was 10.5. Addition of 20 mg/mL of GdL gave a final pH of 3.5 while addition of 2 molar equivalents of Ca^{2+} ions with respect to 2NapLG gave a final pH of 10.0. Images were collected under cross-polarized (top) and non-polarized (bottom) light. (b) shows the 2NapLG crystals within the 2NapFF gel. Being within a gel network makes collecting high quality images of the crystals a challenge. Scale bars represent 300 μm.

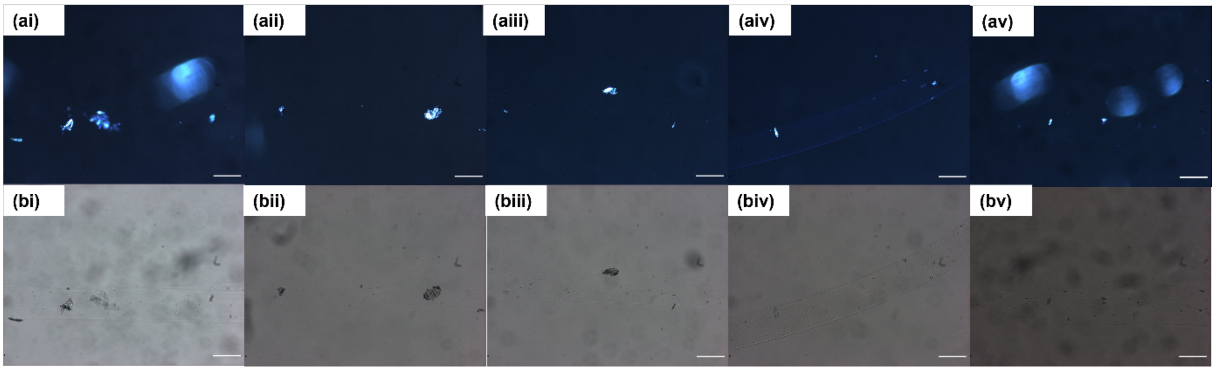

Figure S10. Images collected from optical microscopy of 2NapLG crystals within supramolecular gel noodles under (a) polarized and (b) non-polarized light. Scale bars represent 300 μm.

Kinetics of self-assembly *via* reduction in pH

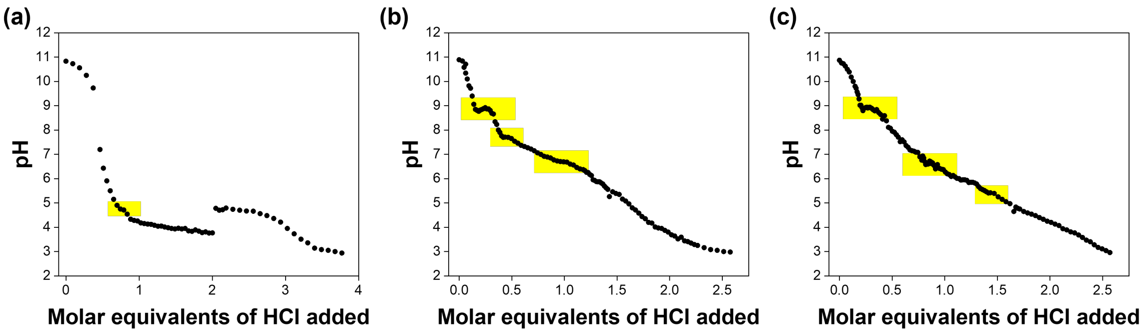

Figure S11. pK_a titrations of (a) 2NapLG 2.5 mg/mL, (b) 2NapFF (5 mg/mL) and (c) 2NapLG 2.5 mg/mL:2NapFF 5 mg/mL. The yellow shading highlights the plateaus from which the pK_a values were extrapolated.

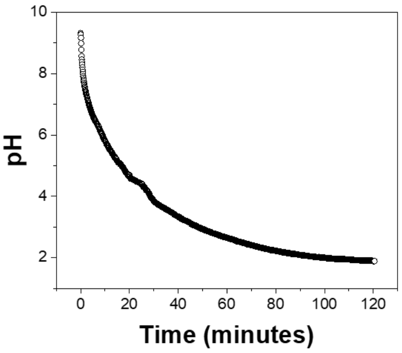

Figure S12. pH values collected from a sample of 2NapLG 2.5mg/mL:2NapFF 5 mg/mL (2 mL sample volume in a 7 mL Sterilin vial) going from a Ca^{2+} gel to a GdL gel on addition of excess GdL (solid) to the top of the sample.

The fact that 2NapLG alone can form crystals on addition of $CaCl_2$ but when mixed with 2NapFF addition of $CaCl_2$ results in formation of a stable gel with no visible crystals is evidence for co-assembly between 2NapFF and 2NapLG between specific pH values; presumably the incorporation of the 2NapLG molecules in the assembled structures makes them unavailable for crystal formation. After addition of GdL, the pH drops to ~3.5 where self-sorting allows 2NapLG to form crystals. The plateau in pH log collected during the transition from a Ca gel to a GdL gel (Figure S12) at pH ~4.2 matches the pK_a of 2NapLG (Figure S11). The formation of crystals/removal of 2NapLG molecules from the bulk gel network causes the network to shrink and expel the solvent, a process known as syneresis. Syneresis is not observed in the noodles, where crystals were formed at significantly higher pH. We suspect the smaller proportion of 2NapLG molecules being involved in crystal formation prevents disruption of the gel network.

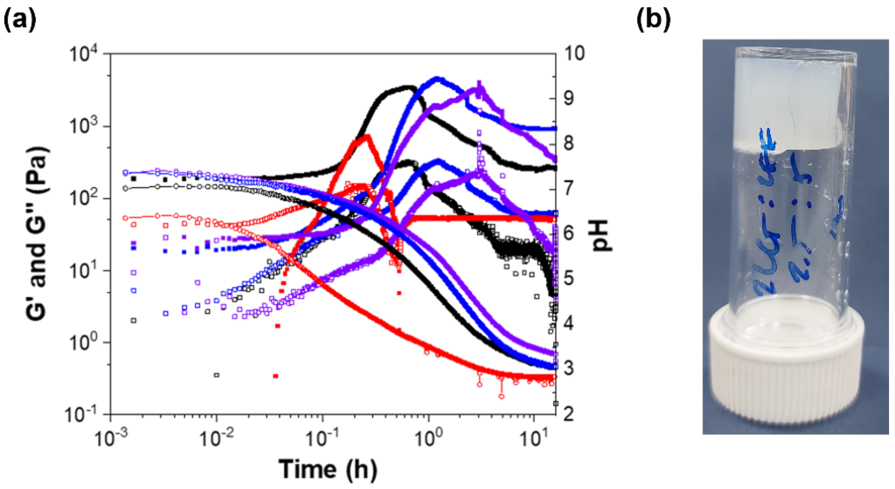

Figure S13. (a) Time sweeps (G′ = filled squares, G″ = empty squares) and pH logs (pH = empty circles) recorded from samples composed of 2NapLG 2.5 mg/mL:2NapFF 2.5 mg/mL. The samples were prepared in 7 mL Sterilin vials with a sample volume of 2 mL. Self-assembly was triggered using GdL (20 mg/mL). The experiments were performed at 25 °C (red), 15 °C (black), 10 °C (blue) and 5 °C (purple). Reducing the temperature reduces the rate of crystal formation and thereby the time taken for the gel to collapse. (b) Photograph showing a syneresed 2NapFF gel with entrapped 2NapLG crystals.

PXRD

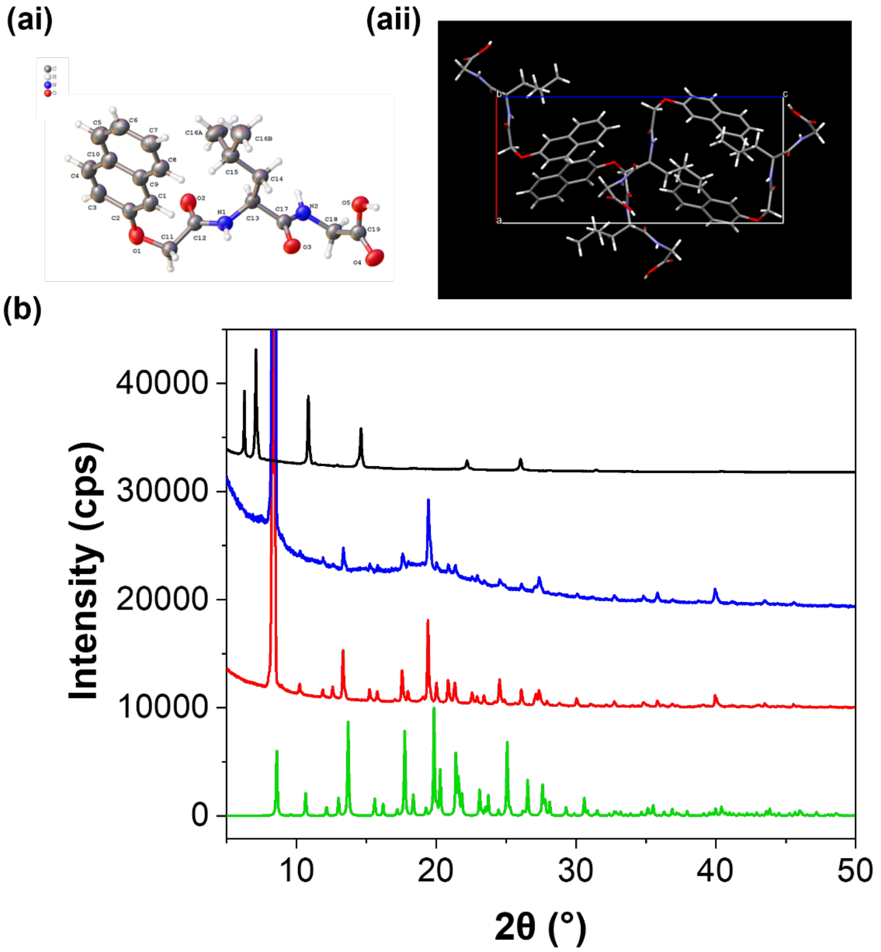

Figure S14. (ai) Single crystal structure and (ii) crystal packing of 2NapLG obtained from crystals prepared from a solution of 2NapLG alone in aqueous conditions on a reduction in pH (from 10.5 to ~3.5) using GdL. (b) Comparison of the single crystal (green) and powder X-ray diffraction patterns obtained from 2NapLG crystals formed alone (red) and in the presence of 2NapFF (blue) in aqueous conditions on a reduction in pH (from 10.5 to ~3.5) using GdL showing the absence of any polymorphism in when the crystals are formed in conjunction with a 2NapFF gel. A PXRD pattern was also obtained from crystals formed from 2NapLG alone in aqueous conditions on addition of 2 molar equivalents of $CaCl_2$ (200 mg/mL, aqueous solution) (black). The pH of the medium following crystal formation was 8.9. These crystals gave a different pattern to those formed by a reduction in pH. However, we were unable to obtain a single crystal diffraction pattern from these crystals and were therefore unable to confirm whether polymorphism was taking place. No crystals are obtained on addition of $CaCl_2$ to 2NapLG 2NapFF multicomponent systems. Co-assembly of 2NapLG and 2NapFF at high pH prevents crystal formation. The single crystal structure details have been deposited, CCDC 2223449, and are available free of charge from the Cambridge Crystallographic Data Centre https://www.ccdc.cam.ac.uk/structures

PhOLL as a second example

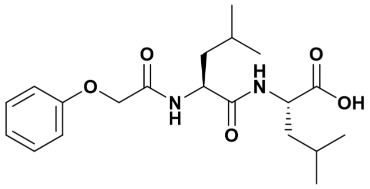

Figure S15. Chemical structure of PhOLL.

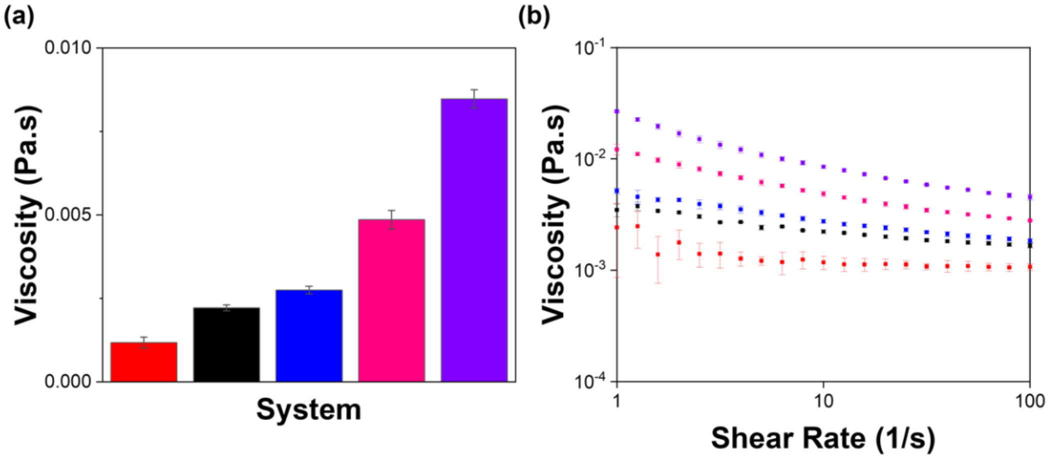

Figure S16. (a) Average viscosity values recorded at a shear rate of 10 s^{-1} for each system in H2O at pH 10.5; PhOLL 2.5 mg/mL (red), 2NapFF 2.5 mg/mL (black), PhOLL 2.5 mg/mL:2NapFF 2.5mg/mL (blue), 2NapFF 5 mg/mL (pink) and PhOLL 2.5 mg/mL:2NapFF 5 mg/mL (purple). Measurements were recorded at temperatures of 25 °C at pH 10.5. Data was collected in duplicate with a fresh sample for each repeat. The error bars show the standard deviation between the samples.

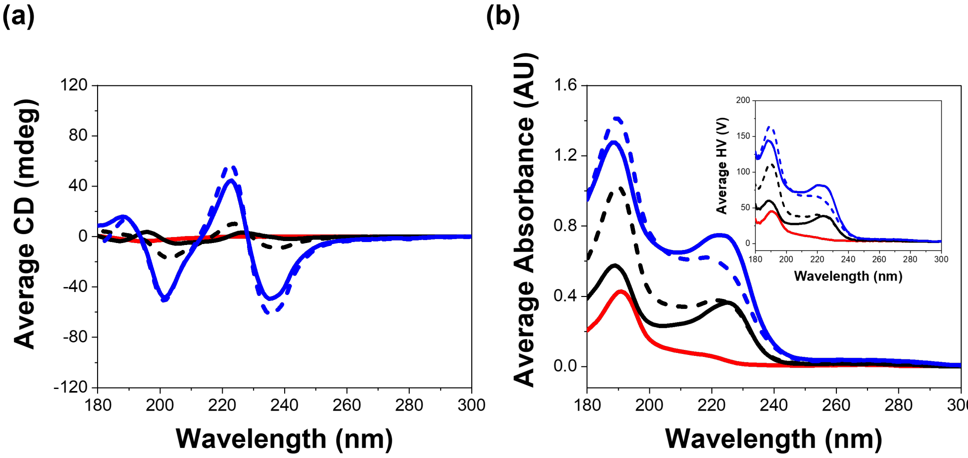

Figure S17. (a) CD spectra and (d) absorbance spectra collected from samples containing PhOLL 2.5 mg/mL alone (red), 2NapFF 2.5 mg/mL alone (black), PhOLL 2.5 mg/mL:2NapFF 2.5 mg/mL (blue), 2NapFF 5 mg/mL alone pink, PhOLL 2.5 mg/mL:2NapFF 5 mg/mL (purple) in H_2O at pH 10.5. All data was collected in triplicate and averaged.

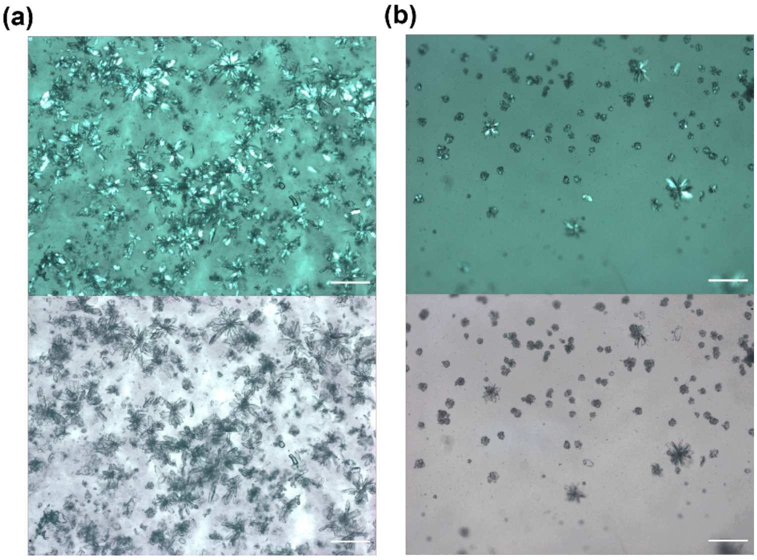

Figure S18. Crystals formed from aqueous solutions of (a) PhOLL alone and (b) PhOLL and 2NapFF formed by a reduction in pH using GdL as a trigger. The starting pH of all solutions was 10.5. Addition of 20 mg/mL of GdL gave a final pH of 3. Images were collected under cross-polarized (top) and non-polarized (bottom) light. Scale bars represent 300 µm.

Sample	PhOLL (2.5 mg/mL) 2FF (2.5 mg/mL)	PhOLL (2.5 mg/mL) 2FF (5 mg/mL)
**A Scale**	2.09 x10-4	0.0014
Scale error	*2.86 x10-6*	*1.85 x10-6*
**Background**	0.0034	0.0042
**Radius**	13	16.27
Radius error	-	*0.038*
**Radius polydispersity**	0.125	0.25
**Thickness**	21	18.83
Thickness error	-	-
**Length**	5000	5000
**B scale**	6.31 x10-5	-
B scale error	*3.42 x10-6*	-
**B power**	1.78	-
B power error	*0.012*	-
**Reduced chi2**	**5.56**	**6.29**

Table S2. Fitting parameters obtained from SasView model fitting of the SANS data. Parameter errors are fitting errors. The length was set to 5000 Å. The SLD, calculated using the NIST SLD calculator,[42] of PhOLL 2.5 mg/mL:2NapFF 2.5 mg/mL is 2.226 x 10^{-6} Å^{-2}, SLD of PhOLL 2.5 mg/mL:2NapFF (5 mg/mL) is 2.394 x 10^{-6} Å^{-2} and SLD of D_2O (the solvent) is 6.393 x 10^{-6} Å^{-2}. PhOLL 2.5 mg/mL:2NapFF 2.5 mg/mL was best fit to a hollow cylinder model combined with a power law while PhOLL 2.5 mg/mL:2NapFF 5 mg/mL was best fit to a hollow cylinder model (Figure S19); these models provided the best fit by eye and lowest chi2 values.

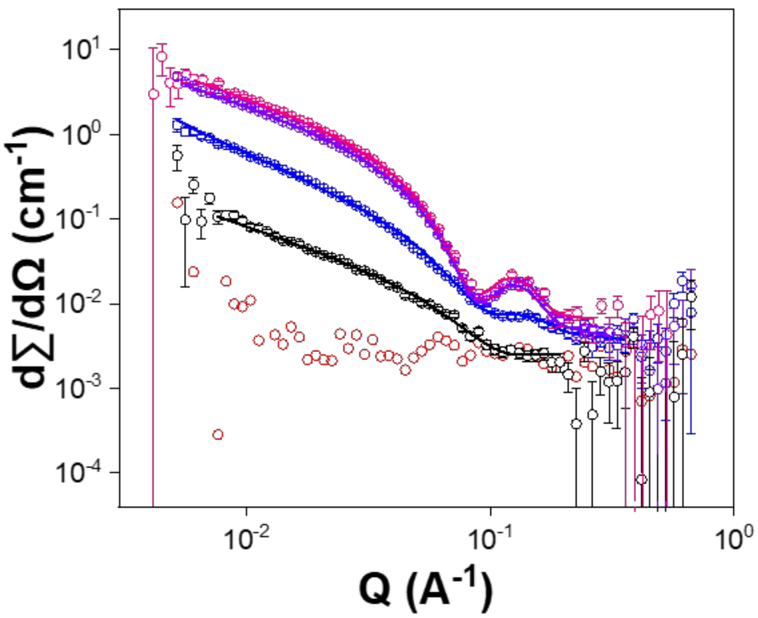

Figure S19. Plots of SANS data (circles) from PhOLL 2.5 mg/mL (red), 2NapFF 2.5 mg/mL (black), PhOLL 2.5 mg/mL :2NapFF 2.5 mg/mL (blue), 2NapFF 5 mg/mL (pink), 2NapLG 2.5 mg/mL:2NapFF 5 mg/mL (purple).

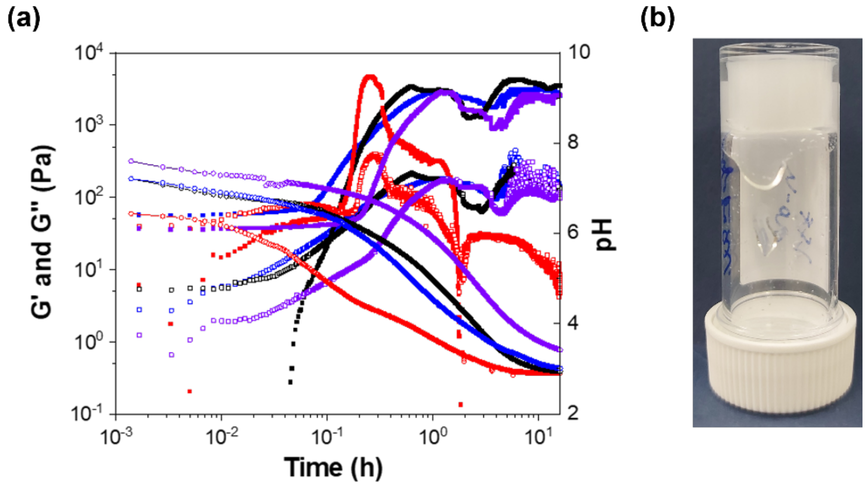

Figure S20. (a) Time sweeps (G′ = filled squares, G″ = empty squares) and pH logs (pH = empty circles). recorded from samples composed of PhOLL 2.5 mg/mL: 2NapFF 2.5 mg/mL. The samples were prepared in 7 mL Sterilin vials with a sample volume of 2mL. Self-assembly was triggered using GdL (20 mg/mL). The experiments were performed at 25 °C (red), 15 °C (black), 10 °C (blue) and 5 °C (purple). (b) Photograph showing a synereSed 2NapFF gel with entrapped PhOLL crystals.

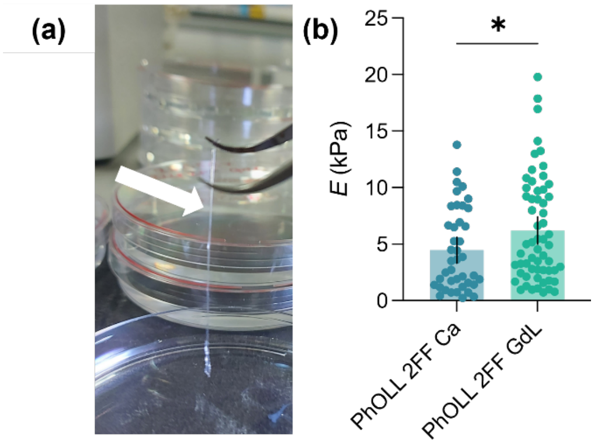

Figure S21. (a) Photograph of a noodle formed from PhOLL (2.5 mg/mL):2NapFF (5 mg/mL) during transfer from the $CaCl_2$ solution in which it was formed to a GdL (40 mg/mL) solution for acidification (noodle highlighted by white arrow for clarity). (b) Results from nanoindentation of noodles composed of 2NapFF (dark green) and PhOLL 2.5 mg/mL:2NapFF 5 mg/mL (light green). Mean ± 95% CI, n=42 for PhOLL 2FF Ca, n=61 for PhOLL 2FF GdL obtained from at least two separate noodles, *p<0.05, two-tailed Mann-Whitney test.
